# New-onset aortic dilatation in the population: a quarter-century follow-up

**DOI:** 10.1007/s00392-022-02086-z

**Published:** 2022-08-26

**Authors:** Cesare Cuspidi, Rita Facchetti, Michele Bombelli, Gino Seravalle, Guido Grassi, Giuseppe Mancia

**Affiliations:** 1 Department of Medicine and Surgery, University of Milano-Bicocca and University Milano-Bicocca Milano, Milan, Italy; 2 IRCSS Istituto Auxologico Italiano, Milan, Italy

**Keywords:** Aortic root dilatation, Echocardiography, General population, Left ventricular mass

## Abstract

**Background:**

Aortic size tends to increase with aging but the extent of this dynamic process has not been evaluated in long-term longitudinal population-based studies. We investigated the incidence of new-onset aortic root (AR) dilatation and its principal correlates among middle-aged adults over a 25-year time period.

**Methods:**

A total of 471 participants with measurable echocardiographic parameters at baseline and after a 25-year follow-up were included in the analysis. Sex-specific upper limits of normality for absolute AR diameter, AR diameter indexed to body surface area (BSA) and to height were derived from healthy normotensive PAMELA participants.

**Results:**

New AR dilatation occurred in 7.4% (AR/BSA), 9.1% (AR/height) and 14.6% (absolute AR), respectively. According to the AR/height index, the risk of new dilation was similar in men and women. As for echocardiographic parameters, baseline AR diameter emerged as a key predictor of AR dilation, regardless of the diagnostic criteria and the 10-year change in LVMI was positively associated to new AR/height dilatation. No significant relationship was observed between baseline office and ambulatory systolic/diastolic blood pressure or their changes over time with incident AR dilatation. Baseline and the 25-year change in 24-h pulse pressure were negatively related to new AR dilatation.

**Conclusions:**

The incidence of AR dilatation from mid to late adulthood occurs in a small but clinically relevant fraction of participants and is unaffected by both office and out-office BP. It is significant related to baseline AR diameter and to the 25-year change in LVMI. Our data suggest that echocardiography performed in middle-aged individuals of both sexes may identify those at increased risk of future AR dilatation; moreover, preventing LVH may reduce the risk of progressive AR enlargement.

**Graphical abstract:**

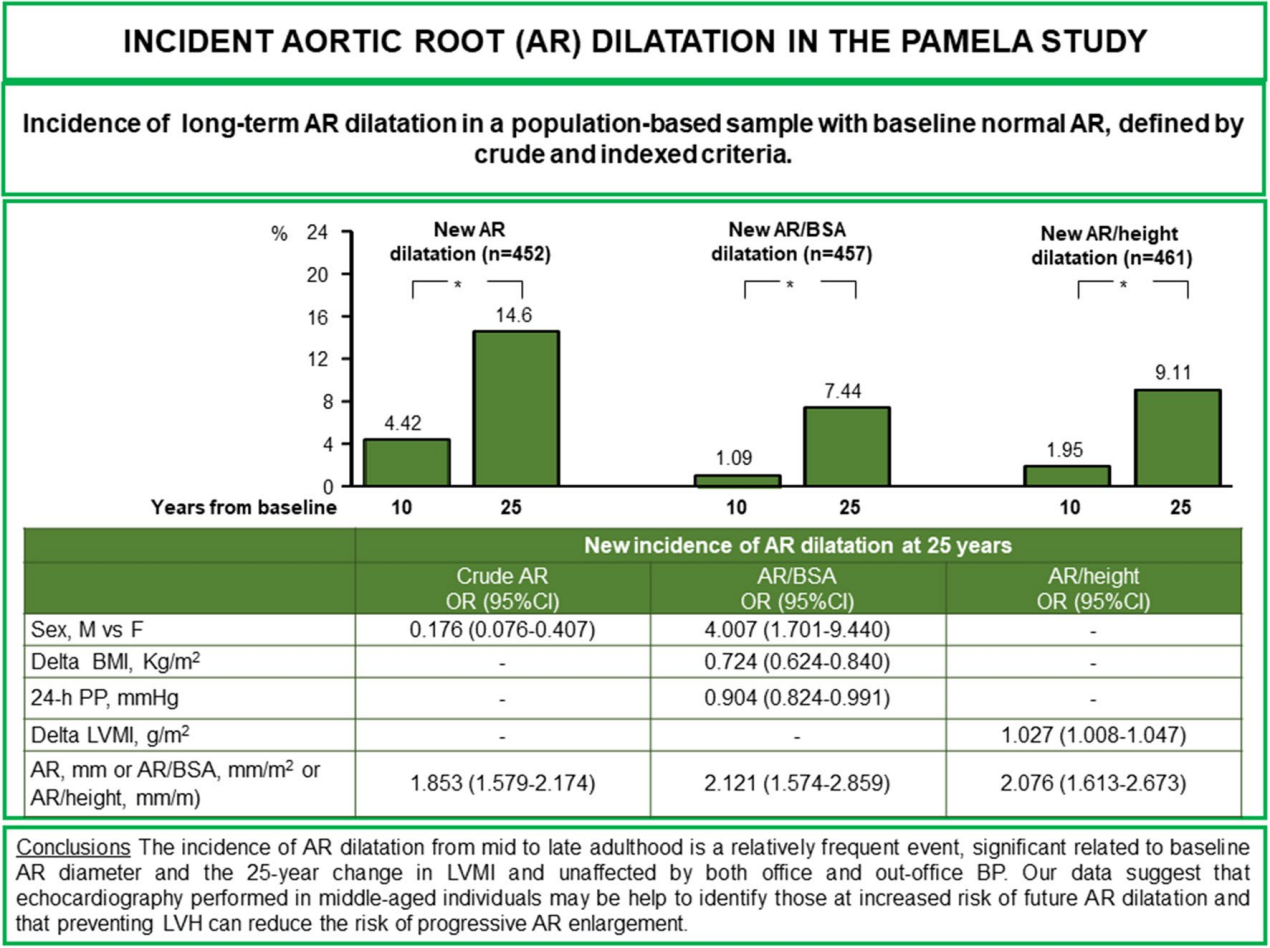

**Supplementary Information:**

The online version contains supplementary material available at 10.1007/s00392-022-02086-z.

## Introduction

Transthoracic echocardiography is routinely used to measure the diameter of aortic root (AR) and proximal aortic segments and thus to identify aortic aneurysms or dilatations in genetic (i.e. Marfan’s and Ehlers-Danlos syndromes) or atherosclerotic diseases [1]. Unlike most echocardiographic measures of cardiac structure [2–5], however, the value of aortic diameter for the stratification of cardiovascular risk in populations free from overt aortic disease is still poorly defined due to the fact that the association of aortic diameter with cardiovascular events and death relies on few longitudinal studies carried out in heterogeneous populations, based on different definitions of the aortic phenotypes and characterized by relatively short follow-ups [6–10]. These are the main reasons why the 2018 European Society of Cardiology/European Society of Hypertension (ESC/ESH) guidelines did not include aortic diameter among the markers of cardiac damage to be considered in hypertensive patients [5]. Furthermore, although it is well known that ascending, thoracic and abdominal aortic diameters increase with aging [11, 12], a limited knowledge exists on the prevalence, correlates and time-related changes of aortic dilatation [3–15]. In the Pressioni Arteriose Monitorate e Loro Associazioni (PAMELA) study, echocardiographic and clinical data (including office ambulatory and home blood pressure, BP) have been collected at the start and after 10 and 25 years in an Italian population sample with an average initial age of 42 years. Based on these data, we were able to assess the long-term incidence of new-onset AR dilation in individuals from middle to old age over a quarter of century.

### Methods

The PAMELA Study was performed in an original sample of 3,200 subjects aged from 25 to 74 years representative of the population of Monza (a town near Milan, Italy) for sex, age and other characteristics. The study was conducted in accordance with the Declaration of Helsinki and approved by the Ethics committee of the San Gerardo University Hospital, Monza (Italy). Informed consent was obtained from all subjects.

As described in detail elsewhere [16]**,** participants were invited to the outpatient clinic of the San Gerardo University Hospital of Monza in the morning of a working day (Monday to Friday), following an overnight fast and abstinence from alcohol and smoking. Data collection included medical history, weight, height, abdominal circumference, standard blood examinations, office, home and 24-h (h) ambulatory (A) blood pressure (BP) and transthoracic echocardiography. Office BP was measured three times with the subject in the sitting position, using a mercury sphygmomanometer and taking the 1^st^ and 5^th^ Korotkoff sounds to identify systolic (S) and diastolic (D) values, respectively. An oscillometric device was used in the third survey (Takeda TM-2430 A&D). In order to measure ABP, subjects were fitted with an ABP monitoring device (Spacelabs 90,207, Issaquah, WA, US) set to obtain automated oscillometric BP and heart rate (HR) readings every 20 min over 24 h. Subjects were asked to pursue their normal activities during the monitoring period, holding the arm still at time of BP readings, going to bed not later than 11.00 p.m. and arising not before 7.00 a. m. Subjects were also asked to self-measure BP at home from the arm contralateral to the one used for ABP measurement, using a validated semiautomatic oscillometric device (model HP 5331, Philips), and a cuff size appropriate to the individual’s arm circumference, at approximately 7 pm and 7am. Advise was given to stay for 5 min in the sitting position and to avoid smoking and alcohol consumption for at least half an hour before measurements. On either occasion, one home BP measurement was obtained at the first survey and two BP measurements at the remaining ones (see below). Participants were again contacted from 2001 to 2003, i.e. after a mean time interval of 10.8 ± 0.5 years and from 2017 to 2018, i.e. after a mean time interval of 25.3 ± 0.5 years; those willing to be re-examined were asked to attend the San Gerardo Hospital for a second and third set of data collection. The same procedures used for the first survey were used for the following surveys except for the doubling of the morning and evening home BP measurements.

### Echocardiography

Transthoracic echocardiographic data were collected according to standard procedures, as previously reported [17]. In brief, M-mode and two-dimensional echo examinations were carried out with a commercially available instrument (Acuson 128 CF, Computer Sonography, Samsung Medison EKO 7). End-diastolic (d) and end-systolic (s) LV internal diameters (LVID), interventricular septum (IVS) thickness and posterior wall (PW) thickness were measured off-line from two-dimensionally guided M-mode tracings recorded at 50–100 cm/s speed, during at least three consecutive cycles. LV mass was estimated using the corrected ASE method: 0.8x(1.04x[(IVSd + LVIDd + PWTd)^3^-LVIDd^3^]) + 0.6 and normalized to body surface area. (BSA). LV hypertrophy (LVH) was defined as LV mass index (LVMI) equal to or higher than 115 g/m^2^ in men and 99 g/m^2^ in women [18, 19]. AR diameter was measured at the level of Valsalva’s sinuses by M-mode tracings, under two-dimensional control, as the maximal distance between the leading edges of anterior and posterior aortic root wall at end diastole (inner edge to inner edge methodology). Sex-specific upper normal limits (mean plus 1.96 standard deviation) for absolute AR diameter as well as for AR diameter indexed to BSA and to height were derived from 712 PAMELA participants (414 women, 298 men) after excluding subjects with isolated home or ambulatory hypertension, obesity, diabetes, cardiovascular diseases; these limits were the following: 3.8 cm, 2.1 cm/m^2^, 2.3 cm/m in men and 3.4 cm, 2.2 cm/m^2^, 2.2 cm/m in women, respectively [8]. An additional analysis identified AR dilatation according to the criteria recently recommended by the British echocardiography guidelines [20], i.e. > 21.8 mm/m in men and > 20.7 mm/m in women. Echocardiographic tracings were obtained by two skilled operators and read by a third independent observer: intra-observer coefficient of variation was 0.6% for LVIDd, 3.1% for IVSd thickness, 3.2% for PWd thickness and 2.0% for ARD.

### Data analysis

Statistical analysis was performed by SAS System (version 9.4; SAS Institute Inc., Cary, North Carolina, USA). In each subject, BP and heart rate at the three office visits, morning and evening home values as well as 24 h values were separately averaged. Only participants with a qualitatively adequate echocardiogram and without AR enlargement, significant cardiac valve disease including bicuspid aorta, > 1 + valvular regurgitation, any degree of valvular stenosis or presence of prosthesis were included in the present analysis.

The rate of incidence of new-onset AR was calculated I) for absolute AR diameter, II) AR indexed to BSA; III) AR indexed to height. In each subject, the changes (Δ) of clinical variables were calculated between the final and baseline evaluation. Incident aortic dilatation was also calculated according to the cut-off values reported by the British echocardiography guidelines [20]. Comparisons were made by chi-square test or Mc Nemar test or Student’s t test (paired and unpaired) or Mann–Whitney test. Repeated Measures Analysis of Variance (ANOVA) and Cochran’s Q test were also applied.

Factors independently related with new-onset AR dilatation were identified by a logistic multivariable model (with stepwise selection) where baseline AR, age, sex, BMI, office, home, 24-h SBP, DBP and pulse pressure (PP), LVM index, plasma glucose, serum cholesterol, antihypertensive treatment and their changes over time (i.e. Δ) were considered as independent variables.

AR diameter cut-offs associated with risk of developing AR dilatation were calculated by Youden index. Sensitivity, specificity, negative and positive predictive value were also calculated. Logistic models were used to calculate odd ratio (and relative 95% confidence intervals) of each cut-offs. A p < 0.05 was considered statistically significant.

### Patient and public involvement

Participants and/or the public were not involved in the design, conduct, reporting or dissemination plans and results of this research.

## Results

**Fig. 1:**
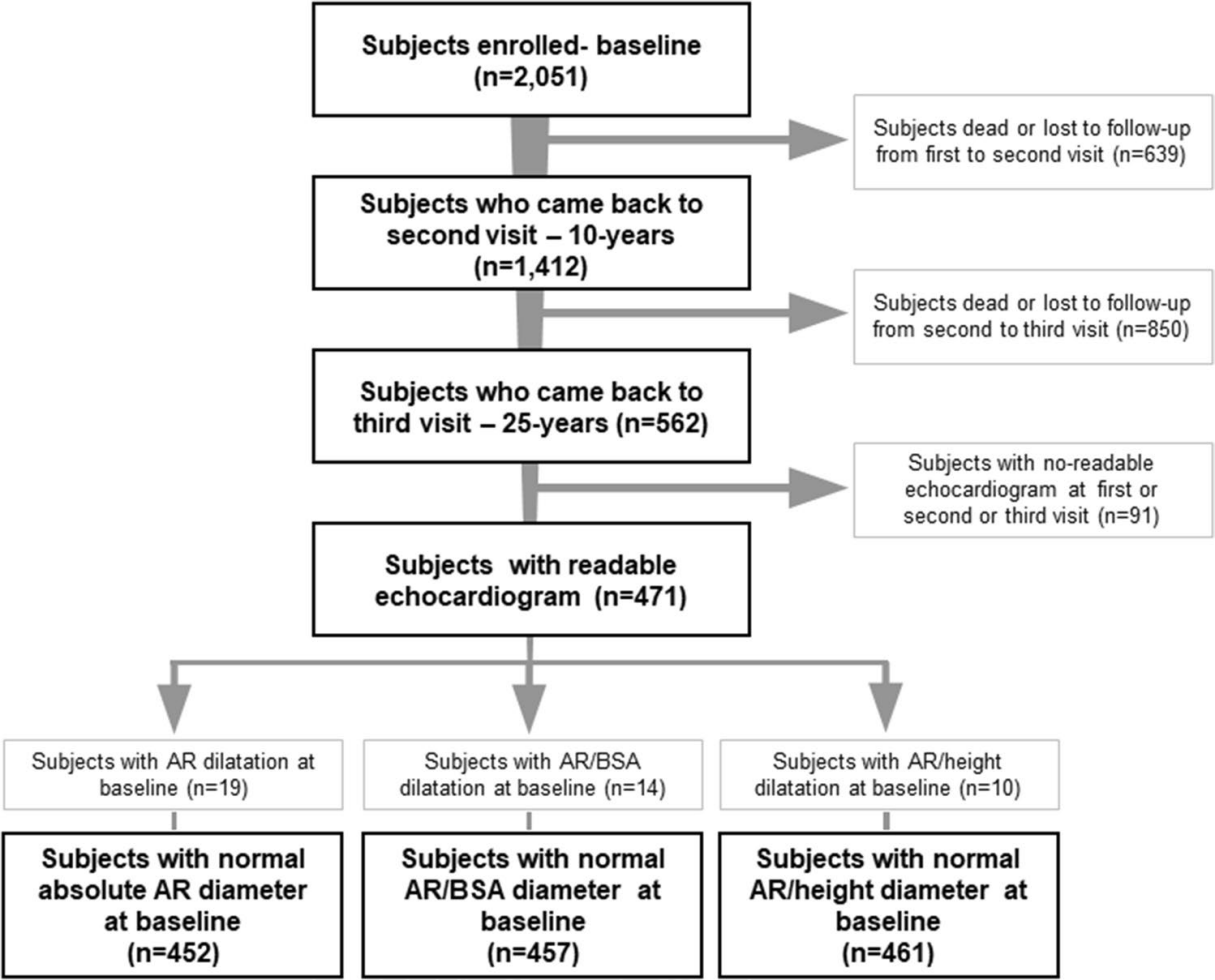
Flowchart showing the selection of participants based on normal absolute aortic root (AR) diameter at baseline with readable echocardiogram at the end of follow-up

The present analysis included all participants without AR dilatation at baseline and with measurable echocardiographic variables of interest in the three surveys. From the original sample of the PAMELA population (3200 subjects), 2051 subjects participated in the first survey, 1412 participated in the second survey and 562 participated in the third survey (Fig. 1). The full set of data (3 valid echocardiographic examinations) was obtained in 452 participants with an initially normal AR size according to non-indexed diameter), 457 participants according to AR/BSA and 461 ones according to AR/height (Fig. 1). Compared to subjects deceased and non-participants, participants included in present analysis were younger, less likely to be overweight or obese and less frequently hypertensive and/or under BP-lowering drugs (data not shown).

Table 1 reports the demographic and clinical data collected during the three surveys in the participants with normal non-indexed AR diameter at baseline (data regarding participants with normal AR/BSA and AR/height are summarized in Supplementary Table 1 and 2).

**Table 1:** Demographic and clinical characteristics of participants with normal non-indexed aortic root diameter at initial, 10-year and 25-year follow-up visit

N = 452	Baseline	10-year	25-year	*p* value
Age, years	40.95 ± 9.61	51.72 ± 9.58	65.7 ± 9.61	–
BSA, m^2^	1.73 ± 0.19	1.77 ± 0.2	1.79 ± 0.21	< 0.0001
Height, cm	165.56 ± 9.45	165.19 ± 9.51	165.29 ± 9.59	0.0236
Weight, kg	66.65 ± 12.69	70.47 ± 13.79	71.69 ± 14.60	< 0.0001
Male, %	218 (48.23%)			–
Office SBP, mmHg	121.77 ± 15.01	129.02 ± 19.48	136.94 ± 18.06	< 0.0001
Office DBP, mmHg	80.35 ± 9.73	82.36 ± 10.58	83.48 ± 8.99	< 0.0001
Office HR, b/min	70.5 ± 9.41	73.1 ± 10.22	70.7 ± 10.13	< 0.0001
24 h SBP, mmHg	116.02 ± 9.06	120.61 ± 11.18	134.1 ± 14.12	< 0.0001
24 h DBP, mmHg	73.02 ± 6.61	75.28 ± 7.45	78.09 ± 7.67	< 0.0001
24 h HR, b/min	76.31 ± 8.28	73.85 ± 8.98	72.3 ± 7.68	< 0.0001
Antihypertensive drugs, %	24 (5.35%)	85 (18.81%)	209 (46.24%)	< 0.0001
ARD, cm	3.02 ± 0.32	3.00 ± 0.38	3.26 ± 0.39	< 0.0001
ARD, cm/m^2^	1.75 ± 0.17	1.70 ± 0.19	1.83 ± 0.21	< 0.0001
ARD, cm/m	1.83 ± 0.17	1.81 ± 0.20	1.97 ± 0.20	< 0.0001
Total cholesterol, mg/dL	212.85 ± 40.56	202.75 ± 33.86	201.89 ± 35.38	< 0.0001
HDL cholesterol, mg/dL	57 ± 15.44	60.74 ± 15.49	59.64 ± 17.5	< 0.0001
Glycemia, mg/dL	86.11 ± 12.13	91.11 ± 22.43	95.31 ± 22.01	< 0.0001
Triglycerides, mg/dL	96.06 ± 54.08	108.11 ± 67.5	108.03 ± 61.25	< 0.0001
LVH, %	22 (5.3%)	78 (17.33%)	63 (14.22%)	< 0.0001
LVMI, g/m^2^	79.65 ± 16.76	89.67 ± 21.57	85.65 ± 20.61	< 0.0001

*ARD* aortic root diameter, *BMI* body mass index, *SBP* systolic blood pressure, *DBP* diastolic blood pressure, *PP* Pulse pressure, *HDL* high density lipoprotein, *HR* heart rate, *LVH* left ventricular hypertrophy, *LVMI* left ventricular mass index, *SBP* systolic blood pressure

**Fig. 2:**
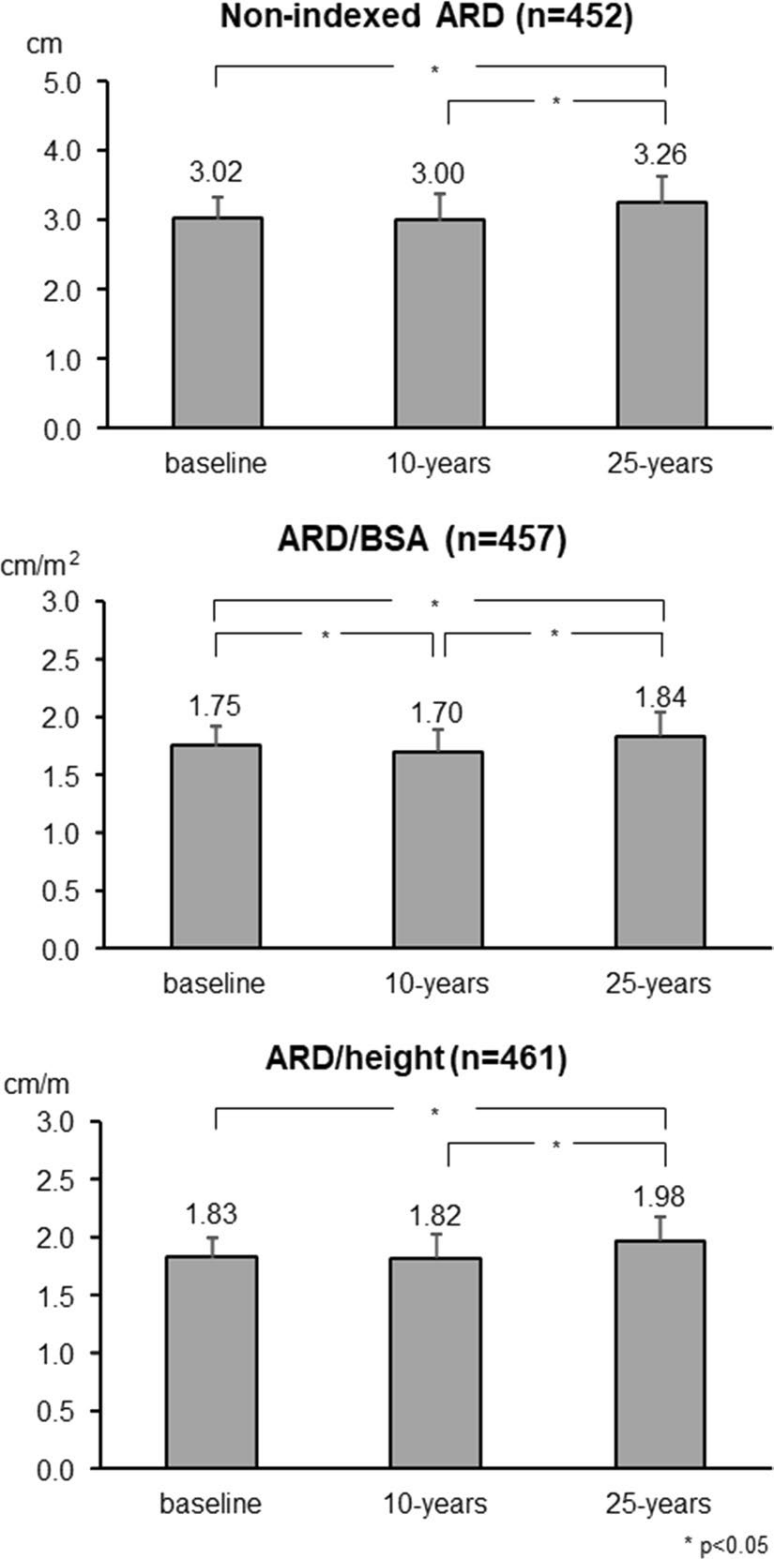
Absolute AR diameter, AR indexed to body surface area (BSA) and AR indexed to height at baseline and after 10- and 25-year follow-up period (mean ± SD)

**Fig. 3:**
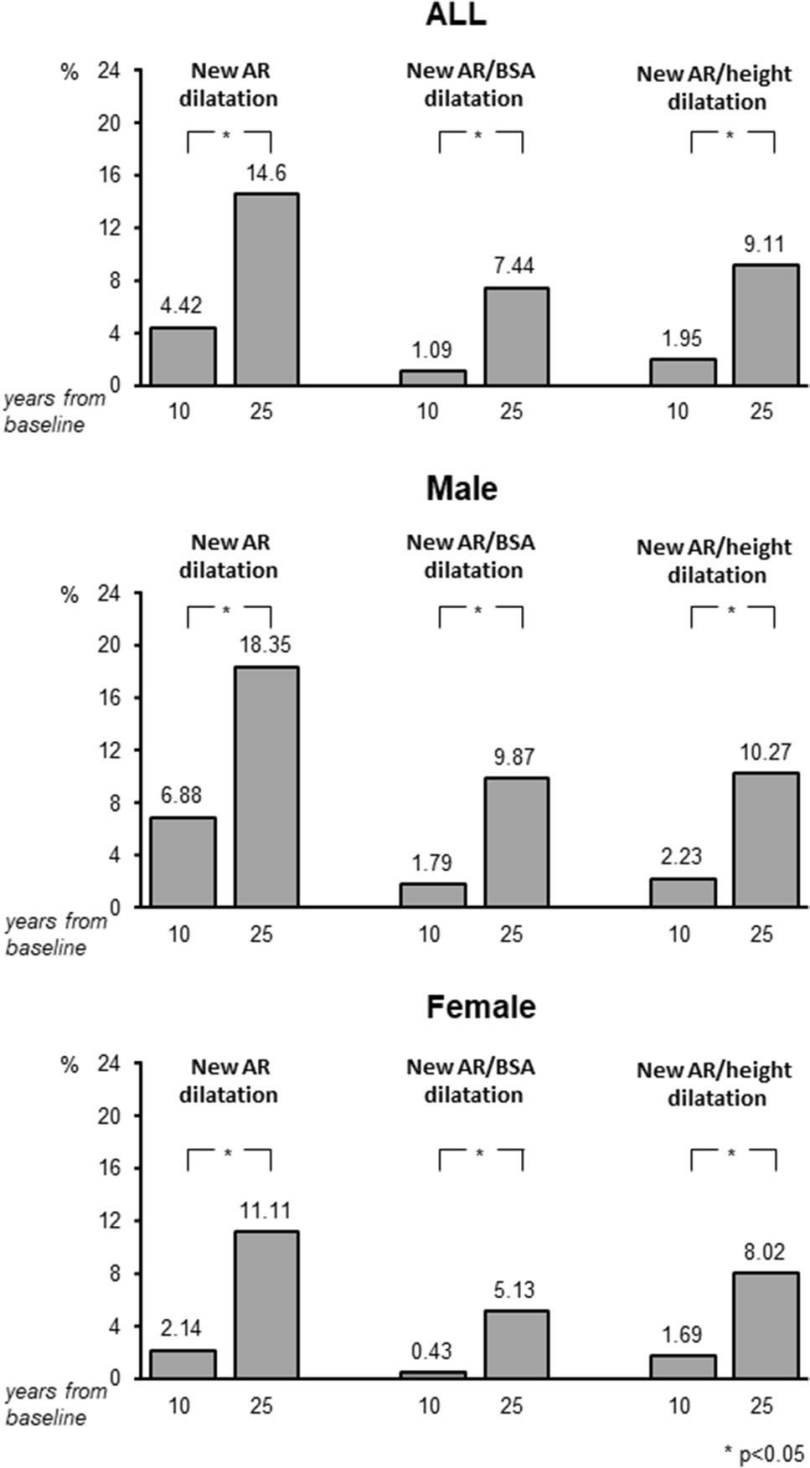
Incidence of new-onset AR dilatation in the whole study population (top panel), in male (middle panel) and female participants (bottom panel), according to absolute AR diameter, AR indexed to body surface area (BSA) and AR indexed to height after 10- and 25-year period of follow-up

BMI, office, 24-h systolic/diastolic BP, blood glucose, total cholesterol, HDL cholesterol, triglycerides, LVMI, prevalence of LVH and antihypertensive treatment all showed a significant progressive increase from the first to the second and third final survey. In contrast, 24-h (but not office) heart rate showed a progressive reduction, whereas compared to the first survey, indexed mean absolute and indexed AR diameters showed little or no changes at the second survey and a consistent increase at the third survey (about 10 and 25 years later, see Methods, respectively). This is also shown for the AR indexed diameters in Fig. 2 and for the incidence of AR dilatation in Fig. 3. Depending on the criterion used, AR dilatation at the 25-year survey occurred in 7.4% (AR/BSA), 9.1% (AR/height) and 14.6% (non-indexed AR diameter) of participants. The corresponding values at the 10-year survey were 1.1%, 2.0% and 4.4%. The much lower incidence of AR dilatation after 10 than after 25 years was seen both in males and females, although after 25 years, new AR dilation was greater in men than in women regardless the diagnostic criteria employed (Fig. 3). The incidence of AR dilatation after 25 years was 19.6% according to the British echocardiography criteria.

**Fig. 4:**
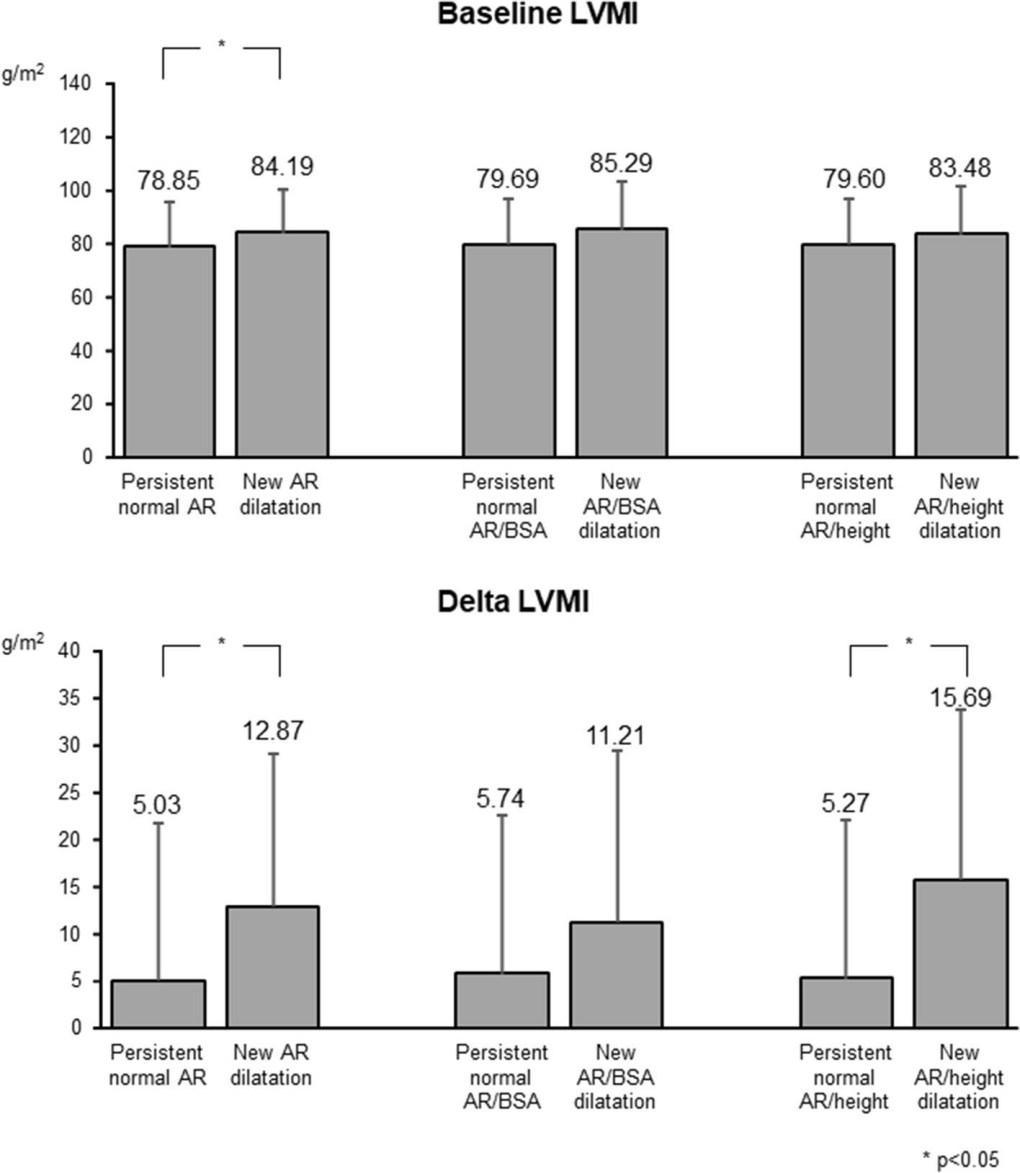
Left ventricular mass index (LVMI) at baseline (top panel) and changes in LVMI during the 25-year follow-up period (bottom panel) in participants with persistent normal AR and in those with new-onset AR dilation according to absolute AR diameter, AR indexed to body surface area (BSA) and AR indexed to height

Table 2 shows the demographic and clinical characteristics of participants who developed AR dilatation (defined by the three diagnostic criteria) and of those with a persistently normal AR diameter at the first survey. Independent of the diagnostic criteria used, participants with AR dilatation were older and more frequently men (non-indexed and AR/BSA criteria). They also had higher office and 24-h systolic and diastolic BP, higher blood glucose values and higher absolute, AR/BSA and AR/height diameter. LVMI was also significantly greater in participants who developed AR dilatation according to the non-indexed criterion. Neither office nor 24-h heart rate values were different in the two groups. As shown in Table 3, there were modest and variable differences in BMI, office DBP and 24-h DBP changes during the 25 years follow-up between participants with dilated AR/BSA and AR/height compared to those with persistently normal AR diameter. The only clear follow-up difference observed between the two groups was a significant greater increase in LVMI in participants developing AR dilatation after 25 years (Fig. 4).

**Table 2:** Clinical characteristics of participants with persistent normal aortic root (AR) diameter and with new AR dilatation categorized in three groups according to absolute AR diameter, AR indexed to body surface area (BSA) and AR indexed to height

	No new AR dilatation	New AR dilatation	*p* value	No new AR/BSA dilatation	New AR/BSA dilatation	*p* value	No new AR/height dilatation	New AR/height dilatation	*p* value
*N*	386	66		423	34		419	42	
Age, years	40.35 ± 9.57	44.45 ± 9.16	0.0013	40.77 ± 9.63	45.8 ± 8.64	0.0033	40.46 ± 9.54	46.92 ± 7.99	< .0001
BMI, Kg/m^2^	24.07 ± 3.5	25 ± 3.01	0.0424	24.36 ± 3.5	23.79 ± 3	0.3601	24.12 ± 3.42	25.34 ± 3.58	0.0299
Male, %	178 (46.11%)	40 (60.61%)	0.0295	201 (47.52%)	22 (64.71%)	0.0537	201 (47.97%)	23 (54.76%)	0.4012
Office SBP, mmHg	121.33 ± 14.7	124.33 ± 16.57	0.1333	121.45 ± 14.86	126.35 ± 15.95	0.0661	121.38 ± 14.63	126.19 ± 17.15	0.0462
Office DBP, mmHg	79.89 ± 9.63	83.03 ± 9.95	0.0151	80.16 ± 9.6	83.47 ± 10.58	0.0555	80.01 ± 9.57	84.1 ± 10.33	0.0092
Office PP, mmHg	41.45 ± 9.2	41.3 ± 10.75	0.9097	41.3 ± 9.43	42.1 ± 8.33	0.3427	41.37 ± 9.34	42.1 ± 10	0.6314
Office HR, b/min	70.86 ± 9.34	68.36 ± 9.57	0.0458	70.42 ± 9.48	69.76 ± 10.44	0.7008	70.56 ± 9.46	68.48 ± 9.63	0.1755
24 h SBP, mmHg	115.79 ± 8.91	117.39 ± 9.86	0.1842	116.05 ± 9.03	115.31 ± 9.06	0.6468	115.88 ± 8.97	117.65 ± 9.71	0.2269
24 h DBP, mmHg	72.62 ± 6.45	75.31 ± 7.12	0.0022	72.94 ± 6.59	74.4 ± 6.5	0.2150	72.76 ± 6.52	76.17 ± 6.7	0.0014
24 h PP, mmHg	43.16 ± 4.87	42.08 ± 4.95	0.0976	43.07 ± 4.88	40.66 ± 4.51	0.0115	43.13 ± 4.88	41.49 ± 4.71	0.0381
24 h HR, b/min	76.42 ± 8.22	75.66 ± 8.64	0.4928	76.33 ± 8.48	74.76 ± 7.94	0.2971	76.24 ± 8.36	76.23 ± 8.91	0.9925
Antihypertensive drugs, %	22 (5.74%)	2 (3.03%)	0.5547	28 (6.62%)	1 (2.94%)	0.7129	25 (6.01%)	2 (4.76%)	1.0000
ARD, cm	2.97 ± 0.3	3.32 ± 0.25	< 0.0001	3.02 ± 0.33	3.28 ± 0.33	< 0.0001	3.01 ± 0.32	3.32 ± 0.31	< 0.0001
ARD follow-up, cm	3.16 ± 0.31	3.83 ± 0.28	< 0.0001	3.23 ± 0.37	3.81 ± 0.4	< 0.0001	3.21 ± 0.35	3.88 ± 0.34	< 0.0001
ARD, cm/m^2^	1.74 ± 0.16	1.82 ± 0.17	0.0001	1.74 ± 0.16	1.89 ± 0.15	< 0.0001	1.74 ± 0.16	1.87 ± 0.17	< 0.0001
ARD follow-up, cm/m^2^	1.8 ± 0.18	2.06 ± 0.18	< 0.0001	1.81 ± 0.18	2.24 ± 0.11	< 0.0001	1.8 ± 0.18	2.17 ± 0.16	< 0.0001
ARD, cm/m	1.8 ± 0.16	1.96 ± 0.14	< 0.0001	1.82 ± 0.17	1.97 ± 0.16	< 0.0001	1.82 ± 0.16	1.99 ± 0.16	< 0.0001
ARD follow-up, cm/m	1.92 ± 0.16	2.27 ± 0.11	< 0.0001	1.95 ± 0.19	2.31 ± 0.15	< 0.0001	1.94 ± 0.17	2.36 ± 0.1	< 0.0001
Total cholesterol, mg/dL	211.62 ± 38.39	220.15 ± 51.33	0.1165	212.65 ± 40.63	215.97 ± 41.2	0.6469	211.66 ± 39.23	222.4 ± 52.28	0.1025
HDL cholesterol, mg/dL	56.99 ± 14.61	57.05 ± 19.78	0.9810	56.47 ± 14.53	59.81 ± 23.07	0.2221	56.94 ± 14.79	55.95 ± 20.75	0.6894
Glycemia, mg/dL	85.67 ± 11.38	88.75 ± 15.71	0.0887	85.85 ± 11.25	91.53 ± 19.83	0.0347	85.64 ± 11.07	91.31 ± 18.73	0.0154
Triglycerides, mg/dL	95.15 ± 54.09	101.48 ± 54.15	0.1462	96.37 ± 54.44	96.47 ± 47.26	0.6421	94.63 ± 52.61	107.71 ± 62.86	0.1078
LVH, %	19 (5.38%)	3 (4.84%)	1.0000	21 (5.34%)	2 (6.25%)	0.6883	21 (5.44%)	2 (5.26%)	1.0000
LVMI, g/m^2^	78.85 ± 16.75	84.19 ± 16.21	0.0204	79.69 ± 16.81	85.29 ± 18.12	0.0722	79.6 ± 16.82	83.48 ± 18.01	0.1781

For abbreviations see Table 1

**Table 3:** Delta follow-up-baseline changes in clinical characteristics of participants with persistent normal aortic root (AR) diameter and with new AR dilatation categorized in three groups according to absolute AR diameter, AR indexed to body surface area (BSA) and AR indexed to height

	No new AR dilatation	New AR dilatation	*p* value	No new AR/BSA dilatation	New AR/BSA dilatation	*p* value	No new AR/height dilatation	New AR/height dilatation	*p* value
*N*	386	66		423	34		419	42	
Delta BMI, Kg/m^2^	1.95 ± 3.23	1.87 ± 3.01	0.8444	2.13 ± 3.25	–0.17 ± 2.84	< .0001	1.98 ± 3.32	1.73 ± 2.76	0.6309
Delta Office SBP, mmHg	15.22 ± 17.32	14.86 ± 16.8	0.8753	14.93 ± 17.47	13.05 ± 16.77	0.5443	15.46 ± 17.59	11.06 ± 14.13	0.1176
Delta Office DBP, mmHg	3.45 ± 12.07	1.24 ± 12.29	0.1696	3.37 ± 12.13	–1.26 ± 10.28	0.0308	3.54 ± 12.26	–1.3 ± 9.52	0.0035
Delta Office HR, b/min	0.06 ± 11.32	1.2 ± 9.51	0.4391	0.31 ± 11.13	–0.19 ± 10.57	0.8017	0.12 ± 11.26	1.77 ± 9.63	0.3020
Delta Office PP, mmHg	11.76 ± 13.39	13.62 ± 13.55	0.2992	11.56 ± 13.67	14.31 ± 11.33	0.2539	11.92 ± 13.71	12.37 ± 11.19	0.8378
Delta 24 h SBP, mmHg	17.85 ± 14.2	19.53 ± 17.96	0.3956	18.14 ± 14.86	15.71 ± 15.23	0.3616	18.41 ± 14.8	14.42 ± 14.75	0.0965
Delta 24 h DBP, mmHg	5.25 ± 9.1	4.03 ± 10.68	0.3292	5.17 ± 9.4	2.14 ± 9.69	0.0713	5.36 ± 9.3	1.34 ± 9.17	0.0078
Delta 24 h HR, b/min	–3.88 ± 8.2	–4.71 ± 7.94	0.4454	–3.97 ± 8.3	–3.26 ± 7.55	0.6319	–3.94 ± 8.1	–4.55 ± 8.74	0.6465
Delta 24 h PP, mmHg	12.6 ± 9.7	15.5 ± 12.26	0.0323	12.96 ± 10.2	13.58 ± 9.07	0.7352	13.05 ± 10.16	13.08 ± 10.11	0.9848
Delta Total cholesterol, mg/dL	–8.31 ± 47.11	–26.77 ± 54.61	0.0045	–11.53 ± 48.91	–18.56 ± 41.07	0.4154	–9.55 ± 47.71	–29.21 ± 55.14	0.0125
Delta Glycemia, mg/dL	8.61 ± 17.43	12.8 ± 27.76	0.2429	9.05 ± 19.26	11.88 ± 23.75	0.4177	8.91 ± 18.64	12.55 ± 26.64	0.3925
Delta LVMI, g/m^2^	5.03 ± 17.35	12.87 ± 21.02	0.0078	5.74 ± 17.89	11.21 ± 20.61	0.1113	5.27 ± 17.49	15.69 ± 20.96	0.0008

For abbreviations see Table 1

### Predictors of new-onset AR dilatation

Logistic models with stepwise selection were constructed to identify baseline (i.e. at the first survey) factors independently associated to incidence of AR dilatation after 25 years. As shown in Table 4, male gender, initial (or baseline) AR/BSA diameter, baseline 24-h PP and the 25-year change of BMI were independently correlated to incident AR/BSA dilatation, whereas for the AR/height dilatation, the independent predictors were baseline AR/height diameter and the 25-year change of LVMI. Baseline AR diameter (1.83** ± **0.17 cm/m), baseline 24-h PP (43.0** ± **4.9 mmHg) and 25 year change of LVMI (6.1** ± **1.81 g/m^2^) were the independent predictors of AR dilatation also with the adoption of the British echocardiography guidelines.

**Table 4:** Multivariable odd ratios (ORs) and 95% confidence intervals (CIs) for new-onset aortic root (AR) dilatation for participants with normal AR diameter at baseline

	Non-indexed AR dilatation				AR/BSA dilatation				AR/height dilatation			
	OR	CI95%		*p* value	OR	CI95%		*p* value	OR	CI95%		*p* value
Sex, M vs F	0.176	0.076	0.407	< .0001	4.007	1.701	9.44	0.0015	–	–	–	–
Delta BMI, Kg/m^2^	–	–	–	–	0.724	0.624	0.84	< .0001	–	–	–	–
24-h PP, mmHg	–	–	–	–	0.904	0.824	0.991	0.0322	–	–	–	–
Delta LVMI, g/m^2^	–	–	–	–	–	–	–	–	1.027	1.008	1.047	0.006
AR, mm or AR/BSA, mm/m^2^ or AR/height, mm/m)	1.853	1.579	2.174	< .0001	2.121	1.574	2.859	< .0001	2.076	1.613	2.673	< .0001

### Additional analyses

To further explore the association between changes in BP over time and the incidence of AR dilatation, we divided the participants with AR dilatation into four subgroups: with persistently normal office BP (i.e. < 140/90 mmHg in all three visits), normal in two out of three visits, normal in one out of three visits, and persistently elevated. We did the same procedure for BP control according to ambulatory criteria (i.e. average 24-h BP < 130/80 mmHg). This categorical classification did not reveal significant differences in AR dilatation (AR/BSA and AR/height) among the various groups (data not shown). Finally, we explored the potential association between abnormal circadian BP pattern and incident AR dilatation. We found that the prevalence of the non-dipping pattern (i.e. systolic nocturnal SBP fall < 10%) at baseline in participants who developed non-indexed AR dilatation did not differ from participants with persistently normal AR diameter (22 vs 25%, *p* = 0.56). This was also the case for new-onset AR/BSA and AR/height dilatation.

Finally, Supplementary Table 3 reports AR diameter cut-offs associated with risk of new-onset AR dilatation.

## Discussion

The present study provides longitudinal data on the changes of aortic diameter and the incidence of AR dilatation trough a 25-year period in a sample of a middle-aged Italian population examined at 10 and 25 years after the initial examination. The most important findings are the following. One, mean AR diameter increased in the population sample over the 25 years observation period, but the increase was not linear throughout this period, i.e. it was minimal after about 10 years from the initial survey and more pronounced after the following 15 years. Two, over the 25-year time interval, new-onset AR dilatation occurred in a small but clinically relevant fraction of participants, the proportion ranging from 7 to 14% depending on the criterion used to define AR dilatation. The highest number of patients diagnosed as having AR dilatation were those in whom AR dimensions were based on crude sex-specific cut-off values, followed by AR cut-offs indexed to height and, lastly, by cut-offs indexed to BSA. Application of the British echocardiography guidelines [20] led to an even greater incidence of AR dilatation, i.e. about one out of five individuals. Three, incident AR dilatation was detected more frequently in men, although the male/female ratio also varied in relation to the diagnostic criterion employed, i.e. from 1.2 (AR/height) to 2.1 (absolute AR). Of note, the association between sex and risk of new-onset AR in the multivariable analysis varied according to the phenotype definition, that is it was higher in women based on crude cut-offs, higher in men based on indexation to BSA and similar in both sexes based on indexation to height. Finally, baseline AR diameter emerged as the key predictor of AR dilation in all multivariable models regardless of definition. In contrast, no other demographic and clinical variable was independently associated with future AR dilation regardless the AR diagnostic criteria applied, including those recommended by the British Society of Echocardiography [20], two exceptions, however, were present. Although incident AR dilatation was not related to baseline office or out-of-office (ambulatory and home) BP, when quantified as AR/BSA, it did show a negative relationship with 24-h pulse pressure at the initial survey. Furthermore, the increase in LVMI observed during the follow-up period showed an independent association with the risk of AR dilatation when AR diameter was indexed to height.

These heterogeneous results obtained in our study about the incidence of AR dilatation in the total population and in the gender-based analysis according to different diagnostic criteria, raise the question about the most appropriate criterion defining this phenotype. No doubt that the use of absolute AR diameter may be misleading, because this measure does not take into account the physiological variability of aortic diameter associated with body dimensions, sex and age [2]).This may result in a substantial overestimation of AR dilatation in individuals with a large body size, thus compromising the predictive value of this parameter for cardiovascular risk stratification and its clinical implications [22]. Concerning the stratification of cardiovascular risk it is noteworthy that in a previous publication of our group, AR/BSA and AR/height, but not absolute AR, were independent predictors of non-fatal and fatal cardiovascular events [8]. In the debate about the more reliable method for normalizing aortic diameter, the Normal Reference Ranges for Echocardiography (NORRE) study and the recent World Alliance of Societies of Echocardiography (WASE) Normal Values Study stated that, compared to BSA indexation, the aortic/height index is superior for the estimating adverse outcomes [1, 23]. Both data-set demonstrated that aortic dimensions are more closely related to height than BSA in all age strata. Another advantage of height is that, unlike weight (which over adult life can vary markedly), height remains fairly constant. This represents a clear advantage for the assessment of temporal changes in AR diameter. Finally, in the present study, the aortic/height index showed that, with only two exceptions (men at higher risk of AR dilatation by aortic BSA index and women at higher risk of AR dilatation according to no- indexed diameter), the risk of AR dilation in the general population was unrelated to gender regardless of the cut-off values (PAMELA and British guidelines). Thus, from our data, the use of aortic to height standardized criteria appear to be preferable for routine clinical practice.

Several other aspects of our study deserve mention. The first aspect is that available data on the incidence of AR dilatation in population-based samples based on follow-up periods greater than 10 years are limited to few studies. The Framingham Heart Study investigators tracked absolute AR diameter changes over 16 years in 3506 individuals in mid to late adulthood and showed that the increase in AR diameter was related principally to older age, male sex, body size and higher diastolic BP [14]. The authors, however, did not report the incidence of AR dilatation as a categorical variable. In the Coronary Artery Risk Development in Young Adults (CARDIA), the changes in absolute aortic dimensions among 3501 young adults were assessed over a 20-year period during which mean AR diameter increased by about 2 mm (from 27.8** ± **4 to 30.7** ± **4 mm, *p* < 0.001) [24]. Unlike the above-mentioned studies, we defined the aortic phenotype more comprehensively using not only the increase in the crude diameter but also the AR increase indexed to BSA and height in order to limit the confounding effects of interindividual differences in body size. The second aspect of our study is that baseline office BP values and their changes over time, even after adjustment for antihypertensive treatment, did not emerge as independent predictors of incident AR dilatation. Furthermore, when changes in BP were treated as categorical variables (i.e. persistently normal BP in all three visits, normal in two out of three visits, normal in one out of three visits, and persistently elevated) failed to show an association with new-onset AR dilatation. This was the case also for baseline BP values and changes in ambulatory and home BP (data not shown). This is a novel finding, as no previous study on AR diameter had included out-of-office BP in the data collection. It is also a novel finding that baseline 24-h PP was negatively related to incident AR dilatation. This extends a previous observation of the Framingham study of an inverse relationship between office PP and changes in AR size from mid to late adulthood [14]. Although counter intuitive and at first sight, this observation is in agreement with the results provided by direct haemodynamic measurements reporting an association between higher PP, increased aortic impedance and reduced rather than increased AR diameter [25]. Finally, we failed to find an association between the non-dipping pattern and incident AR dilatation.

A third aspect concerns again the importance of indexing aortic diameter to measures that help reducing interindividual body size differences. In addition to the above-mentioned advantages, in our study, the use of AR/height index highlighted an independent correlation between the occurrence of AR dilatation and LVMI increase. In this context, the correlation between AR dilatation and age is consistent with previous observations on the age-related combined arterial and ventricular remodeling [26, 27], probably due to age-associated increased collagen and reduced elastin content of the vascular walls, thus enhancing aortic stiffness as well as LV afterload, and favoring LV remodeling and LVH [28–30]. Finally, a further contribution of the present study is the demonstration of the key role of the length of the follow-up in assessing changes in AR diameter over time. In our cohort, the medium-term (10 years) incidence of AR dilatation was low, i.e. less than 5% if defined by the absolute AR diameter and between 1–2% according to AR indexes. This suggests that the findings provided by most studies that assessed the impact of aging on the AR diameter over a follow-up of 10 years or less may not reliably reflect the changes occurring over the course of a lifetime.

Our study has some limitations. Our results refer to a population sample with an initial age of 40 years, with a low prevalence of obesity, diabetes, hypertension and LVH. Thus, extrapolation to populations at cardiovascular high-risk and prevalence of cardiovascular disease should be done with caution. Furthermore, the non-attendance at follow-up of older participants and those who had cardiovascular events may have determined an underestimation of incident AR dilatation**.** It is worth noting that our findings refer only to AR diameter and that they may not reflect the age-related changes of other aortic segments. Last, technical aspects, such as AR diameter measurement based on M-mode echocardiography and use of ultrasound devices with different technical characteristics during the 25-year study, may have influenced our results.

## Conclusions

The present study is the first to provide 25-year longitudinal data on AR dilatation from mid to late adulthood. The incidence of AR dilatation was almost negligible after 10 years of follow-up while became more evident after 25 years; patients exhibiting aortic dilatation ranged from 7 to 15% depending on the diagnostic criteria used to define dilatation. Baseline and changes in office and out-of-office BP failed to show any relationship with AR dilatation; only baseline high-normal values of AR diameter and LVMI increase during the observation period were independent predictors. From a clinical perspective, these findings support the view that echocardiographic examinations performed in middle-aged individuals may be help to identify those at increased risk of future AR dilatation; moreover, measures aimed at preventing LVH may contribute to reduce the risk of progressive AR enlargement.

### Supplementary Information

Below is the link to the electronic supplementary material.

Supplementary file1 (DOCX 16 KB)

Supplementary file2 (DOCX 15 KB)
