# Correction: New-onset aortic dilatation in the population: a quarter-century follow-up

**DOI:** 10.1007/s00392-022-02127-7

**Published:** 2022-11-23

**Authors:** Cesare Cuspidi, Rita Facchetti, Michele Bombelli, Gino Seravalle, Guido Grassi, Giuseppe Mancia

**Affiliations:** 1 Department of Medicine and Surgery, University of Milano-Bicocca and University Milano-Bicocca Milano, Milan, Italy; 2 IRCSS Istituto Auxologico Italiano, Milan, Italy

## Correction: Clinical Research in Cardiology 10.1007/s00392-022-02086-z

The affiliation of the author Gino Seravalle was corrected to ‘IRCSS Istituto Auxologico Italiano, Milan, Italy’. The original article has been corrected.

