# Supplementary material for: New-onset aortic dilatation in the population: a quarter-century follow-up
Source: Clin Res Cardiol. 2022 Aug 26;112(11):1529–40. doi: 10.1007/s00392-022-02086-z (PMC10584747; doi:10.1007/s00392-022-02086-z)
Supplement: Supplementary file 1 — Supplementary file1 (DOCX 16 KB) [file 392_2022_2086_MOESM1_ESM.docx]

**Supplementary table 1**

Demographic and clinical characteristics of all participants at initial and 25-year follow-up visit with normal AR/BSA diameter at baseline.

| **N=457** | **Baseline** | **10-years** | **25-years** | **p-value** |
| --- | --- | --- | --- | --- |
| Age, years | 41.14±9.64 | 51.9±9.61 | 65.89±9.64 |  |
| BSA, m^2^ | 1.74±0.19 | 1.78±0.2 | 1.79±0.21 | <0.0001 |
| Height, cm | 165.78±9.45 | 165.4±9.51 | 165.52±9.57 | 0.0216 |
| Weight, kg | 67.13±12.75 | 70.93±13.81 | 72.25±14.95 | <0.0001 |
| Male, % | 223 (48.8%) |  |  | - |
| Office SBP, mmHg | 121.81±14.98 | 129.46±19.54 | 136.61±18.12 | <0.0001 |
| Office DBP, mmHg | 80.41±9.7 | 82.57±10.55 | 83.44±8.93 | <0.0001 |
| Office HR, b/min | 70.37±9.54 | 72.99±10.15 | 70.62±10.13 | <0.0001 |
| 24h SBP, mmHg | 116±9.03 | 120.7±11.17 | 133.91±14.14 | <0.0001 |
| 24h DBP, mmHg | 73.05±6.59 | 75.47±7.51 | 77.97±7.63 | <0.0001 |
| 24h HR, b/min | 76.21±8.44 | 73.76±8.96 | 72.26±7.75 | <0.0001 |
| Antihypertensive drugs, % | 29 (6.35%) | 90 (19.69%) | 216 (47.26%) | <0.0001 |
| ARD, cm | 3.04±0.34 | 3.02±0.4 | 3.28±0.4 | <0.0001 |
| ARD , cm/m^2^ | 1.75±0.17 | 1.7±0.19 | 1.84±0.21 | <0.0001 |
| ARD , cm/m | 1.83±0.17 | 1.82±0.21 | 1.98±0.21 | <0.0001 |
| Total cholesterol, mg/dL | 212.89±40.64 | 202.7±34.44 | 200.87±35.54 | <0.0001 |
| HDL cholesterol, mg/dL | 56.72±15.32 | 60.54±15.29 | 59.35±17.56 | <0.0001 |
| Glycemia, mg/dL | 86.27±12.16 | 91.23±22.39 | 95.51±22.33 | <0.0001 |
| Triglycerides, mg/dL | 96.38±53.89 | 108.64±68.08 | 108.24±61.17 | <0.0001 |
| LVH, % | 23 (5.41%) | 80 (17.58%) | 64 (14.32%) | <0.0001 |
| LVMI, g/m^2^ | 80.11±16.95 | 89.91±21.57 | 86±20.95 | <0.0001 |

ARD: Aortic root diameter; LVH: Left Ventricular Hypertrophy (LVMI BSA≥99(F)/114(M)
