# Supplementary material for: New-onset aortic dilatation in the population: a quarter-century follow-up
Source: Clin Res Cardiol. 2022 Aug 26;112(11):1529–40. doi: 10.1007/s00392-022-02086-z (PMC10584747; doi:10.1007/s00392-022-02086-z)
Supplement: Supplementary file 2 — Supplementary file2 (DOCX 15 KB) [file 392_2022_2086_MOESM2_ESM.docx]

**Supplementary table 3**

Cut-off of aortic root diameter (ARD) associated with risk of developing aortic dilatation and relative odd ratio (OR).

|  | AUC | Cut-off* | SE | SP | NPV | PPV | N | N New ARD | OR (95%CI) p-value |
| --- | --- | --- | --- | --- | --- | --- | --- | --- | --- |
| Absolute ARD | 0.8135 | 3.19 | 0.77 | 0.76 | 0.95 | 0.35 |  |  |  |
| ARD, cm≤3.19 |  |  |  |  |  |  | 308 | 15 | ref |
| ARD, cm>3.19 |  |  |  |  |  |  | 144 | 51 | 10.71 (5.76-19.93) p<0.0001 |
|  |  |  |  |  |  |  |  |  |  |
| ARD/BSA | 0.7490 | 1.89702 | 0.68 | 0.81 | 0.97 | 0.23 |  |  |  |
| ARD, cm/m^2^≤1.89702 |  |  |  |  |  |  | 356 | 12 | ref |
| ARD, cm/m^2^>1.89702 |  |  |  |  |  |  | 101 | 22 | 7.98 (3.79-16.81) p<0.0001 |
|  |  |  |  |  |  |  |  |  |  |
| ARD/height | 0.7823 | 1.93567 | 0.69 | 0.76 | 0.96 | 0.22 |  |  |  |
| ARD, cm/m≤1.93567 |  |  |  |  |  |  | 327 | 10 | ref |
| ARD, cm/m>1.93567 |  |  |  |  |  |  | 125 | 24 | 7.12 (3.56-14.21) p<0.0001 |
|  |  |  |  |  |  |  |  |  |  |

*Youden Index cut-off; AUC=Area under the curve, SE=sensitivity, SP=specificity, NPV=negative predictive value, PPV=positive predictive value, OR=odd ratio.
